# Emprego do retalho inguinal nas lesões traumáticas da mão

**DOI:** 10.1055/s-0045-1814123

**Published:** 2025-12-30

**Authors:** Edie Benedito Caetano, João José Sabongi Neto, Luiz Angelo Vieira, Vinicius Santos Bueno, Victor Hugo Monfrin Torres, Karen Cristina Barbosa Chaves

**Affiliations:** 1 Departamento de Cirurgia, Faculdade de Ciências Médicas e da Saúde, Pontifícia Universidade Católica de São Paulo, Sorocaba, SP, Brasil

**Keywords:** arteria ilíaca, enxerto de pele, lesões da mão, lesões dos tecidos moles, retalhos cirúrgicos, hand injuries, iliac artery, skin graft, soft tissue injuries, surgical flaps

## Abstract

**Objetivo:**

Analisar o emprego do retalho inguinal no tratamento de lesões traumáticas com perda tecidual, não muito extensas, na região do punho e mão.

**Métodos:**

Foram realizados 14 retalhos da região inguinal para a reconstrução de lesões traumáticas das mãos. A faixa etária da amostra foi de 21 a 56 anos, e todos os pacientes eram do sexo masculino.

**Resultados:**

A sobrevida completa do retalho foi registrada em todos os membros. Em sete pacientes houve complicações: em dois, necrose parcial, sendo necessário desbridamento do retalho, e a área foi revestida com enxerto de pele. Em quatro casos, ocorreu necrose marginal, que cicatrizou tardiamente de forma espontânea. A soltura parcial na área receptora foi registrada em um caso, na primeira semana, sendo necessário completar a sutura.

**Conclusão:**

Mesmo com o advento das técnicas microcirúrgicas, o retalho inguinal continua a ser um método bom e seguro de reparo de lesões com perda tecidual da mão, com ou sem perda tecido ósseo.

## Abstract (en)

**Objective:**

To analyze the use of the groin flap in the treatment of traumatic injuries that are not very extensive, with tissue loss in the wrist and hand region.

**Methods:**

We performed 14 flaps from the inguinal region to reconstruct traumatic hand injuries. The age of the sample ranged from 21 to 56 years, and all patients were male.

**Results:**

Complete flap survival was recorded in all limbs. In seven patients, there were complications: in two, partial necrosis, for which flap debridement was necessary, and the area was covered with a skin graft. In four cases, marginal necrosis occurred, which later healed spontaneously. Partial loosening at the receiving area was observed in one case in the first week, requiring complete suturing.

**Conclusion:**

Even with the advent of microsurgical techniques, the groin flap remains a good and safe method to repair hand injuries with tissue loss, with or without loss of bone tissue.

## Introdução

Uma cobertura adequada nas lesões complicadas de partes moles da mão é um problema bastante comum e desafiador para cirurgiões de mão, especialmente quando estruturas como ossos, tendões, nervos e vasos sanguíneos são expostas. Na maioria dos casos, essa lesões resultam de traumas graves causados por serras circulares, prensas e maquinaria utilizada na mecânica e na agricultura, por exemplo. Vários retalhos que são úteis para revestir essas lesões têm sido descritos, 1 2 e um deles, utilizado no tratamento das lesões de partes moles das mãos com perda tecidual, é o retalho inguinal, descrito inicialmente por MacGregor e Jackson 3 em 1972 e amplamente utilizado até os dias atuais. 3 4 5 6

Um retalho ideal para revestir lesões com perda tecidual deveria apresentar baixo risco de complicações operatórias, não ter cicatriz visível na área doadora, não apresentar déficit funcional, fornecer quantidade e qualidade adequadas de tecido e ter um pedículo vasculonervoso consistente, de comprimento adequado e fácil de ser dissecado. Embora esse local doador ainda não tenha sido descrito, o retalho inguinal apresenta muitas vantagens, como anatomia confiável, pouco volume, fácil adaptação à área receptora e baixa morbidade na área doadora, podendo, ainda, ser empregado como retalho osteocutâneo pela inclusão da crista ilíaca. Por todos esses motivos, o retalho inguinal continua a ser amplamente utilizado, devido à sua simplicidade, segurança e versatilidade. 3 4 5 6 7 8 9 10 11 12 13 Uma desvantagem deste procedimento consiste na necessidade de manter a mão junto à região inguinal durante o período de cicatrização, ou seja, por cerca de 3 semanas, o que é bastante incômodo para o paciente. Ademais, pelo fato de apresentar um pedículo curto, seu emprego como retalho livre é difícil, além do fato de este retalho não ter potencial para inervação motora para ser utilizado como retalho funcional. 5 6 7 8

Revestir o local da lesão com tecidos moles apropriados é fundamental para a melhora das funções das extremidades. 1 2 5 Uma cobertura ótima deve ser estável, durável e capaz de suportar grandes demandas de trabalho, deve preservar a mobilidade articular e ter aparência esteticamente aceitável, mas sempre privilegiando a função. 5 6 7 8

O objetivo deste trabalho foi apresentar nossa experiência e avaliar nossos resultados com o retalho inguinal na reconstrução de perdas teciduais não muito extensas das mãos associadas ou não a perda de tecido ósseo.

## Materiais e Métodos

### Anatomia

**Fig. 1:**
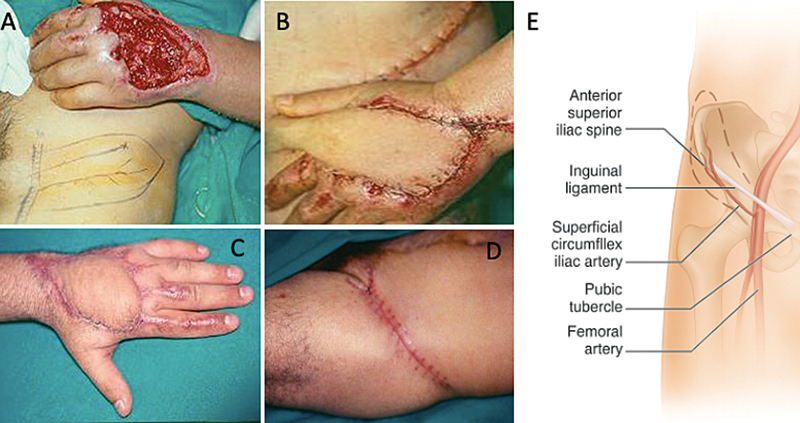
( **A** ) Desenho do retalho na área doadora; ( **B** ) retalho suturado na área receptora; ( **C** ) resultado na área receptora; ( **D** ) resultado na área doadora; e ( **E** ) desenho esquemático com a marcação dos elementos anatômicos que envolvem o retalho.

O retalho inguinal é um retalho fasciocutâneo, suprido pela artéria circunflexa ilíaca superficial; o retorno venoso é dado pela veia de mesmo nome, que se posiciona ao lado da artéria. A artéria e veia circunflexas ilíacas superficiais originam-se da artéria e da veia femorais, cerca de 2 cm distalmente ao ligamento inguinal, o qual une o tubérculo púbico à espinha ilíaca anterossuperior, separando o abdômen do membro inferior. A artéria e veia circunflexas ilíacas superficiais posicionam-se 2 cm distalmente ao ligamento inguinal e correm paralelamente a ele, sendo este o centro do retalho. 3 4 5 6 7 14 Com relação ao comprimento do retalho inguinal, ele pode se estender vários centímetros além da espinha ilíaca anterossuperior, mas a largura não deve ultrapassar 10 cm. Medialmente, o retalho não deve se estender além da artéria femoral, que deve ser localizada por palpação e marcada; é mais seguro completar a dissecção do retalho na distância de 2 cm lateralmente à artéria ( Fig. 1 ).

### Material clínico

**Fig. 2:**
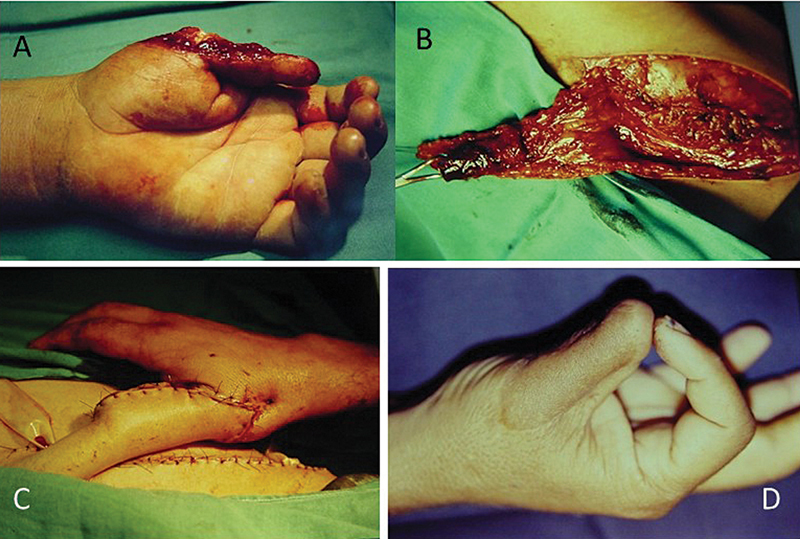
( **A** ) Perda osteocutânea do polegar; ( **B** ) retalho inguinal com inclusão óssea da crista ilíaca; ( **C** ) retalho suturado na região receptora; e ( **D** ) resultado final.

Foram realizados 14 retalhos fasciocutâneos da região inguinal, sendo que em 3 foi necessário incluir um segmento ósseo da crista ilíaca ( Figs. 2 3 4 ). Os retalhos foram utilizados para a reconstrução das mãos e dos dedos entre 2001 e 2017. A faixa etária da amostra foi de 21 a 56 anos, sendo todos os pacientes do sexo masculino. A dimensão das lesões variou de 5 × 8 cm a 10 × 18 cm. Os procedimentos cirúrgicos foram realizados de 2 a 8 dias após a ocorrência da lesão. O pedículo só foi desvinculado da área doadora entre 21 e 24 dias. Na maioria casos, as lesões foram resultantes de acidentes de trabalho, causadas por prensas, máquinas industriais e serras circulares. Em dois pacientes ocorreu lesão dos tendões extensores ( Figs. 5 6 ), e, em quatro, lesão óssea, como fraturas e perda de tecido ósseo ( Figs 2 4 6 ). Em um caso, houve perda tecidual das superfícies palmar e dorsal dos dedos médio e anular ( Fig. 7 ).

**Fig. 3:**
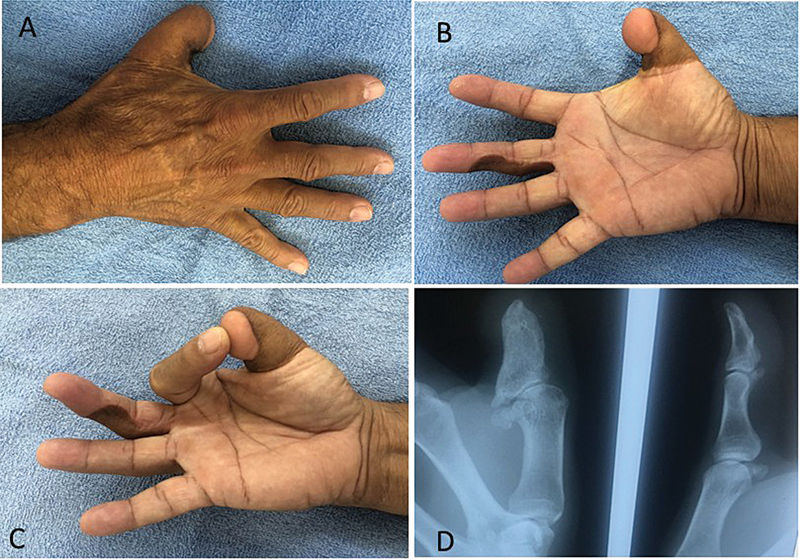
( **A** ) Reconstrução do polegar amputado com retalho osteocutâneo da região inguinal; ( **B** , **C** ) retalho neurovascular em ilha do dedo médio para o polegar; e ( **D** ) radiografia do retalho ósseo do dedo reconstruído ao lado do polegar normal.

**Fig. 4:**
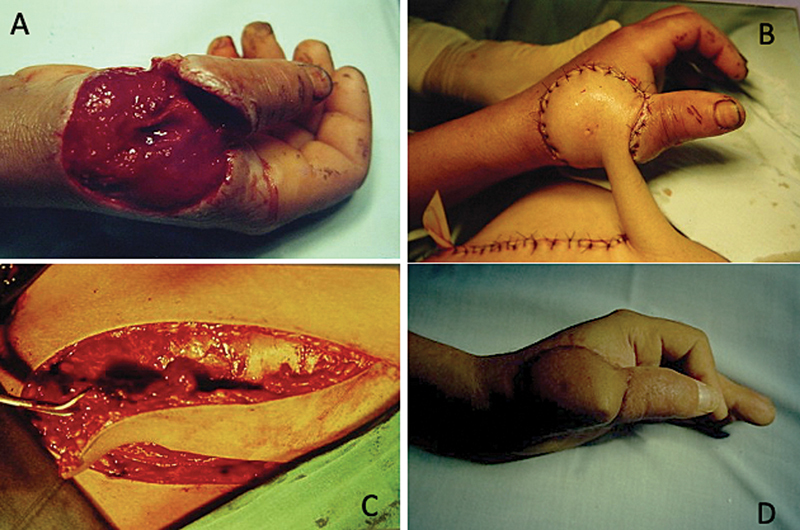
( **A** ) Perda osteocutânea do primeiro metacarpiano; ( **B** ) retalho osteocutâneo com inclusão da crista ilíaca; ( **C** ) retalho osteocutâneo; e ( **D** ) resultado final.

**Fig. 5:**
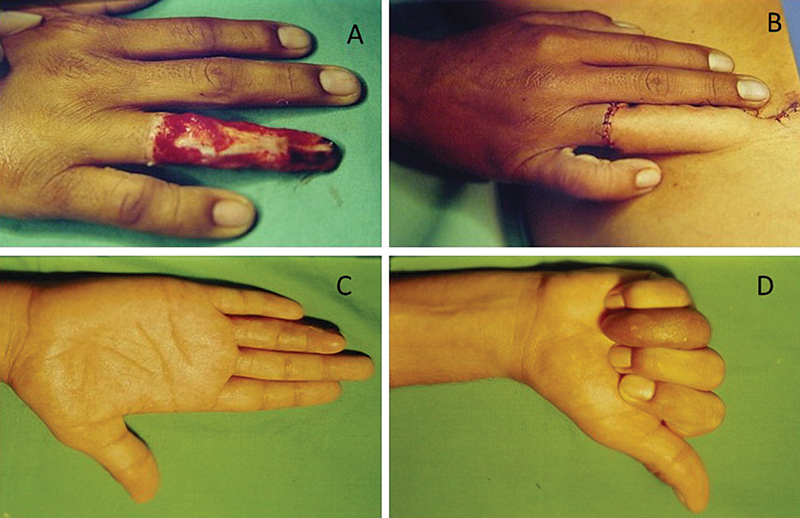
( **A** ) Lesão no dorso do dedo anular; ( **B** ) retalho da região inguinal; ( **C** ) aspecto com extensão dos dedos; e ( **D** ) aspecto com flexão dos dedos.

**Fig. 6:**
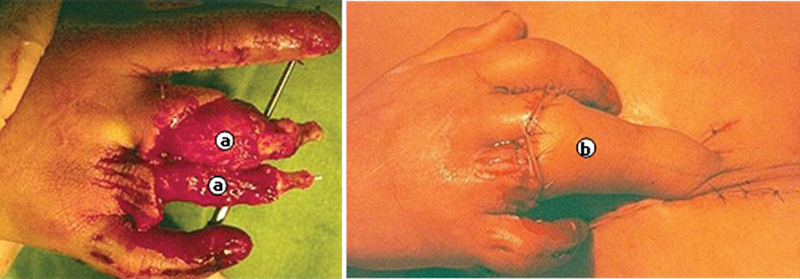
( **A** ) Lesão dos dedos médio e anelar com perda de tecido; e ( **B** ) cobertura com retalho cutâneo inguinal.

**Fig. 7:**
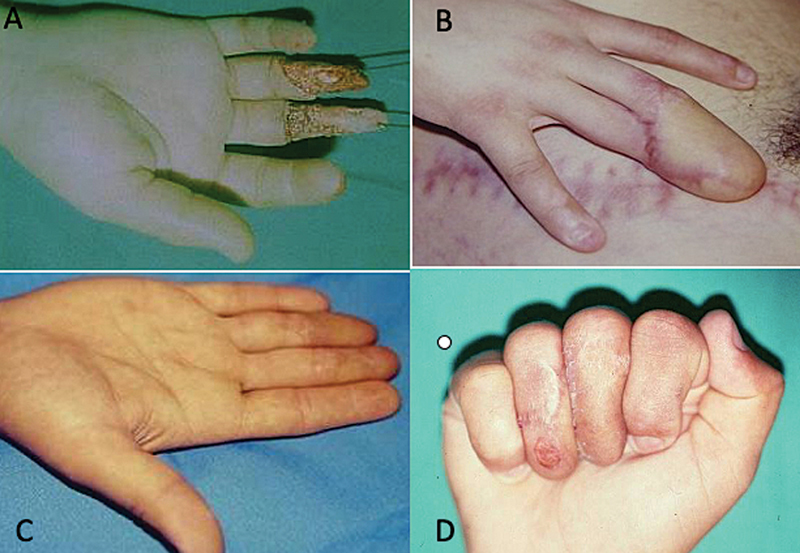
( **A** ) Perda tecidual das superfícies palmar e dorsal dos dedos médio e anular; ( **B** ) áreas doadora e receptora do retalho; ( **C** ) separação dos dedos em extensão; e ( **D** ) aspecto com flexão digital.

### Procedimento cirúrgico

O paciente foi colocado em decúbito dorsal. O retalho foi demarcado de acordo com a necessidade da área receptora. Foram incluídos a pele, o tecido subcutâneo e a fáscia. Em quatro pacientes com perda óssea do polegar, um segmento da crista ilíaca foi incluído no retalho. A inclusão da fáscia faz parte do procedimento, pois os vasos (artéria e veia circunflexas ilíacas superficiais) posicionam-se sobre ela. A dissecção deve ser cuidadosa, com o uso de uma magnificação com lupa e eletrocautério. Após o fechamento da área doadora com leve tensão, foi concluído o procedimento de cobertura da área receptora. Com o pedículo suficientemente solto, pode-se formar um tubo a partir dele a fim de deixar apenas uma área mínima exposta ( Fig. 2 ). Como o retalho só seria desvinculado após 3 semanas e, neste período, os pacientes devem manter a mão junto à virilha, a contenção foi realizada com um enfaixamento do membro junto ao corpo. Não foi necessária a aplicação de fixador externo em nenhum dos casos. Todos os pacientes receberam alta no dia seguinte ao procedimento, e foram devidamente orientados a manter a mão nas proximidades da área doadora.

Este estudo obteve a aprovação pelo Comitê de Ética em Pesquisa institucional, sob o número CAAE: 83985818.7.0000.5373.

## Resultados

**Fig. 8:**
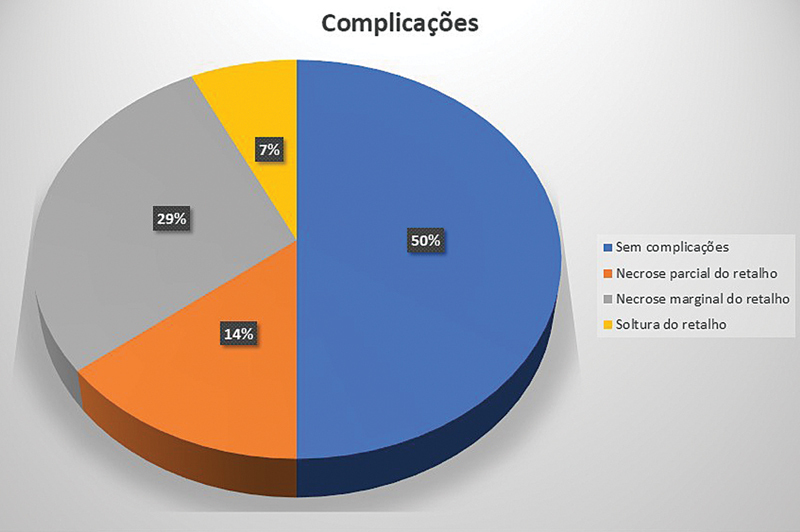
Gráfico com resultados sobre as complicacoes.

Em 7 dos 14 membros, não ocorreram complicações pós-operatórias, com cicatrização primária e completa tanto das áreas doadoras quanto receptoras do retalho. A necrose completa do retalho não foi registrada em nenhum membro. Em sete, houve complicações: necrose parcial ocorreu em dois casos, sendo necessário desbridamento do retalho, e a área foi revestida com enxerto de pele; em 4 casos, ocorreu necrose marginal, que cicatrizou tardiamente de forma espontânea; a soltura parcial na área receptora foi registrada em um caso, na primeira semana, sendo necessário completar a sutura ( Fig. 8 ). Em nenhum dos casos houve comprometimento do retalho pela retirada da contenção por parte dos pacientes, antes de o retalho ser desvinculado.

Os resultados tardios do tratamento foram avaliados em 10 pacientes com média de 24 semanas da cirurgia. Dor nas mãos foi relatada pela maioria dos pacientes, principalmente naquelas lesões resultantes de acidentes de trabalho. Alguns pacientes apresentaram certa limitação da flexão ou da extensão digital. Alguns pacientes também informaram dor na região doadora, principalmente após esforços ou caminhada longa, mas a limitação da mobilidade no membro inferior não ocorreu em nenhum dos pacientes avaliados.

## Discussão

Neste estudo, apresentamos o resultado de 14 retalhos da região inguinal para a reconstrução de lesão das mãos e dos dedos entre 2001 e 2017. Em três pacientes com perda óssea do polegar, um segmento da crista ilíaca foi incluído no retalho. A inclusão da fáscia faz parte do procedimento, pois os vasos (artéria e veia circunflexas ilíacas superficiais) posicionam-se sobre ela. A faixa etária da amostra foi de 21 a 56 anos, sendo todos os pacientes do sexo masculino. A dimensão das lesões variou de 5 × 8 cm a 10 × 18 cm. Os procedimentos cirúrgicos foram realizados de 2 a 8 dias após a ocorrência da lesão. O pedículo só foi desvinculado da área doadora entre 21 a 24 dias, e em 9 dos 14 membros não ocorreram complicações pós-operatórias, com cicatrização primária e completa tanto das áreas doadoras quanto receptoras do retalho. A necrose completa do retalho não foi registrada em nenhum membro. Em sete, houve complicações: necrose parcial ocorreu em dois casos, sendo necessário desbridamento do retalho e a área foi revestida com enxerto de pele; em 4 casos, ocorreu necrose marginal, que cicatrizou tardiamente de forma espontânea; e soltura parcial na área receptora foi registrada em um caso, na primeira semana, sendo necessário completar a sutura.

Żiluk 5 apresentou o resultado do tratamento com retalho inguinal de 31 pacientes com perda tecidual no membro superior, sendo 28 do sexo masculino e 3, do feminino. Ao todo, 23 pacientes tiveram cicatrização primária sem problemas nas áreas doadoras e receptora. Em oito pacientes foram registradas complicações: em um, ocorreu a necrose total do retalho; em três, necrose marginal, que necessitou de enxerto de pele adicional após o retalho ser separado da área doadora; em dois pacientes, os retalhos estavam parcialmente desvinculados da área doadora, e foi necessário o emprego de enxerto de pele; e dois pacientes tiveram retardo de cicatrização na área doadora. O autor 5 relata que as vantagens do retalho inguinal superam as desvantagens, e que resultados do estudo mostram que os retalhos inguinais são úteis para reconstruções de perdas teciduais no membro superior.

Naala et al. 8 relataram os resultados do emprego do retalho inguinal em 85 pacientes com perdas de partes moles no membro superior, 78 do sexo masculino e 7 do feminino, com idade média de 27 anos. Em 99% dos casos, houve boa integração do retalho, e em 1 paciente ocorreu a perda total do retalho. Os desfechos foram satisfatórios em sua maioria, com bons resultados funcionais. No entanto, complicações foram registradas em 22 dos 85 pacientes. Necrose marginal ocorreu em 10 membros. Necrose parcial do retalho que necessitou de reparo com enxerto de pele ocorreu em 6 membros, e infecção no local, em quatro membros. O descolamento do retalho com necessidade de nova fixação cirúrgica aconteceu em dois pacientes. Segundo os autores, 8 apesar de terem sido desenvolvidas técnicas mais recentes de reconstrução, o retalho inguinal continua a ser um método bom e seguro de reparo de lesões com perda tecidual nos membros superiores.

Hayashi et al. 10 relataram o emprego do retalho inguinal em perdas de partes moles em um membro reimplantado, com o retalho inguinal revestindo as faces dorsal e palmar do antebraço. O retalho cicatrizou sem complicações, com resultado funcional satisfatório.

Abdelrahman et al. 11 relataram uma modificação do método do retalho inguinal, com seu emprego sem a inclusão da fáscia. Estudos anatômicos revelaram que a artéria circunflexa ilíaca superficial é suficiente para que um segmento relativamente grande do retalho receba fornecimento adequado de sangue sem a inclusão da fáscia. Naala et al. 8 apresentaram os resultados do tratamento em 77 pacientes com perda tecidual dos membros superiores: o retalho com inclusão da fáscia foi usado em 49 membros, e sem a inclusão da fáscia em 28 membros. Os resultados do tratamento foram satisfatórios nos dois grupos, e os números de complicações do tratamento foram baixos em ambos os grupos.

Jabaiti et al. 12 relataram os resultados do emprego do retalho inguinal em 34 pacientes, sendo 31 do sexo masculino e 3, do feminino: as lesões digitais foram as mais frequentes, e ocorreram em 16 membros. A área média da lesão foi de 44 (variação: 12–162) cm ^2^ . Bons resultados foram observados em 29 pacientes; no entanto, em 1, houve necrose total do retalho e em 4, necrose parcial. Todos os pacientes declararam sua satisfação com os resultados do procedimento. Os autores 12 consideram que, mesmo na era da microcirurgia avançada, o retalho inguinal é uma excelente ferramenta para o revestimento em casos de lesões com perda tecidual de médio e grande portes, e que suas vantagens superam suas desvantagens.

Em seu primeiro estudo, Molski et al. 13 relataram os resultados em 97 pacientes com defeitos teciduais ou deformidades cicatriciais nas mãos, incluindo 87 homens e 10 mulheres com idade média de 25 anos. Dependendo dos tamanhos dos defeitos, os retalhos tinham de 7 a 26 cm de comprimento e de 4 a 12 cm de largura. Em 59 pacientes (61%), a cirurgia foi realizada imediatamente após ou poucos dias após a lesão, ao passo que nos 38 pacientes restantes foi tardia.

Marek 15 empregou em oito pacientes o retalho inguinal osteocutâneo para reparar perda óssea dos metacarpianos e falanges. A cicatrização do retalho foi alcançada em todos os pacientes, embora complicações, incluindo infecção da ferida cirúrgica, necrose parcial do retalho e reação inflamatória nas suturas, tenham sido encontradas em 7 membros. Concluiu-se que o retalho osteocutâneo da região inguinal é uma boa solução para perdas associadas de tecido ósseo e de partes moles.

Gupta et al. 16 apresentaram os resultados do emprego do retalho da região inguinal em 25 crianças com 9 anos ou mais que sofreram perda de tecido da mão como resultado de queimaduras elétricas. Na maioria dos casos, as perdas envolveram exposição de tendões e ossos. A cicatrização do retalho foi alcançada em todos os casos, incluindo 2 casos de necrose parcial que necessitaram de cobertura com enxerto de pele. A deiscência da sutura também foi observada na área doadora em 2 crianças. Exames realizados em média 1 ano após a cirurgia mostraram bons resultados funcionais. As crianças estavam bem, e eram capazes de brincar e realizar suas atividades da vida diária com normalidade.

Devarasetty et al. 17 realizaram um estudo com 88 retalhos inguinais pediculados para coberturas de lesões dos membros superiores. Os pacientes tinham uma idade mediana de 35 anos, e foram submetidos a uma média de 4 cirurgias ao longo de todo o tratamento. A complicação mais comumente observada foi rigidez dos dedos. Outras complicações comuns foram perda parcial do retalho (em 38% dos casos) e infecção (em 32%). Esse estudo apresentou uma taxa de perda parcial do retalho maior em comparação à da nossa amostra, que apresentou apenas 14% de perda parcial do retalho. Lesões de alta energia apresentam um risco maior de necessidade de um maior número de procedimentos ao longo de todo o tratamento. A análise não revelou nenhuma diferença significativa nas complicações da ferida com base nas características do paciente ou da lesão.

## Conclusão

O resultado do tratamento das lesões não muito extensas das mãos, com ou sem perda de tecido ósseo, com o emprego do retalho inguinal, foram satisfatórios. Mesmo com o advento das técnicas microcirúrgicas, o retalho inguinal continua a ser um método bom e seguro de reparo de lesões com perda tecidual da mão, com ou sem perda tecido ósseo.
